# Effects of low and moderate refractive errors on chromatic pupillometry

**DOI:** 10.1038/s41598-019-41296-w

**Published:** 2019-03-20

**Authors:** A. V. Rukmini, Milton C. Chew, Maxwell T. Finkelstein, Eray Atalay, Mani Baskaran, Monisha E. Nongpiur, Joshua J. Gooley, Tin Aung, Dan Milea, Raymond P. Najjar

**Affiliations:** 1 Singapore Eye Research Institute, Singapore, Singapore; 2 Singapore National Eye Centre, Singapore, Singapore; 3 Eskisehir Osmangazi University, Faculty of Medicine, Eskisehir, Turkey; 4 The Ophthalmology & Visual Sciences ACP (EYE-ACP), SingHealth and Duke-NUS, Singapore, Singapore; 5 Centre for Cognitive Neuroscience, Programme in Neuroscience and Behavioural Disorders, Duke-NUS Medical School, Singapore, Singapore; 6 Department of Ophthalmology, Yong Loo Lin School of Medicine, National University of Singapore, Singapore, Singapore

## Abstract

Chromatic pupillometry is an emerging modality in the assessment of retinal and optic nerve disorders. Herein, we evaluate the effect of low and moderate refractive errors on pupillary responses to blue- and red-light stimuli in a healthy older population. This study included 139 participants (≥50 years) grouped by refractive error: moderate myopes (>−6.0D and ≤−3.0D, n = 24), low myopes (>−3.0D and <−0.5D, n = 30), emmetropes (≥−0.5D and ≤0.5D, n = 31) and hyperopes (>0.5D and <6.0D, n = 54). Participants were exposed to logarithmically ramping-up blue (462 nm) and red (638 nm) light stimuli, designed to sequentially activate rods, cones and intrinsically-photosensitive retinal ganglion cells. Pupil size was assessed monocularly using infra-red pupillography. Baseline pupil diameter correlated inversely with spherical equivalent (*R* = −0.26, *P* < 0.01), and positively with axial length (*R* = 0.37, *P* < 0.01) and anterior chamber depth (*R* = 0.43, *P* < 0.01). Baseline-adjusted pupillary constriction amplitudes to blue light did not differ between groups (*P* = 0.45), while constriction amplitudes to red light were greater in hyperopes compared to emmetropes (*P* = 0.04) at moderate to bright light intensities (12.25–14.0 Log photons/cm²/s). Our results demonstrate that low and moderate myopia do not alter pupillary responses to ramping-up blue- and red-light stimuli in healthy older individuals. Conversely, pupillary responses to red light should be interpreted cautiously in hyperopic eyes.

## Introduction

Evaluation of the pupillary light response (PLR) using chromatic pupillometry allows for an objective assessment of photoreceptor health in retinal and optic nerve conditions. The afferent pathway governing the PLR originates from intrinsically photosensitive retinal ganglion cells (ipRGCs)^1–4^, which express the photopigment melanopsin (λmax = 479 nm)^5^ and integrate extrinsic inputs from rods (λmax = 505 nm) and S-cones, M-cones and L-cones (λmax = ~426 nm, ~530 nm and ~552 nm respectively)^6^. Using different wavelengths of light, in what has been labelled as chromatic pupillometry, several studies have attempted to evaluate the integrity of inner and outer retinal photoreceptors^7^. Blue-light stimuli have been used to preferentially activate rods at low irradiances and melanopsin at high irradiances, whereas red-light stimuli preferentially activate cones^8–10^.

Studies using chromatic pupillometry have shown promise in detecting and assessing the severity of glaucoma^8,11–13^ and other optic neuropathies^14–16^, as well as retinal dystrophies^17–19^, macular degeneration^20^, and diabetic retinopathy^21^. Besides clinically established ophthalmic and neurologic conditions, other factors, such as ocular biometry, media clarity^22,23^, and refractive error^24,25^ may influence the PLR^26^ or pupil size^27,28^. Evaluating the impact of such variables on the PLR, especially in older adults in whom the prevalence of ocular diseases like glaucoma, macular degeneration and diabetic retinopathy are greater, is essential for a more accurate interpretation of pupillometric findings in health and disease.

The incidence of myopia is increasing worldwide, with a prevalence of 14% to 50% in the United States and Europe^29,30^, and up to 80% in some East Asian countries^31–34^. The prevalence of hyperopia and astigmatism, on the other hand, increases with age, reaching more than 50% between 60 and 80 years of age in some populations^33,35^. The notion of larger pupils in myopic eyes dates back to the 18^th^ century^36^. In later years, using a ruler with a succession of half circles incremented by 0.5 mm, Hirsch and Weymouth reported that myopic subjects had larger pupils compared to emmetropic and hyperopic subjects^27^. Subsequent studies using more reliable electronic pupillometers have either confirmed^28^, or refuted these findings^37,38^. Correspondingly, investigations of the PLR are also controversial, with some investigators showing differences in some pupillometric features^39^, and not others (*i.e*., post illumination pupillary response (PIPR))^24^. To date, the relationship between chromatic pupillometry outcomes and refractive error remains unclear and conflicting findings could be due to inter-protocol differences in photic stimulation regimens or in data processing (*e.g*., normalization of pupil size to baseline).

There is a paucity of studies evaluating the impact of low and moderate refractive errors on features of the pupillary response, especially in older Asian populations, where the prevalence of refractive errors is high. The aim of our study was to bridge this gap, using ramping-up blue and red light paradigms used in chromatic pupillometry for assessing the integrity of retinal photoreceptors, in a healthy population of older Asian participants.

## Results

### Demographics and ocular characteristics of the study participants

Of the 148 participants who took part in this study, the data from 139 were included in our current analyses. The data of 9 participants were excluded from further analyses due to technical difficulties in data collection (*i.e*., missing refraction values or unreliable data due to excessive blinking). Participants were stratified into four groups based on their spherical equivalent^30^: moderate myopia (>−6.0 diopter (D) and ≤−3.0D, range: −5.75D to −3.0D, n = 24), low myopia (>−3.0D and <−0.5D, range: −2.88D to −0.62D, n = 30), emmetropia (≥−0.5D and ≤0.5D, range: −0.5D to 0.5D, n = 31) and hyperopia (>0.5D and <6.0D, range: 0.62 to 4.13D, n = 54). Participants had a median age of 61.0 years (inter-quartile range: 9.5 years; full range 50–75 years), 51 were males (36.7%), and the majority were Chinese (87.1%) (Table 1). Refractive error groups were not different in their distribution of sex or ethnicity. Emmetropes were significantly younger than hyperopes (H3 = 9.23, *P* = 0.03). As expected, the anterior chamber depth (ACD) and axial length (AxL) were different between groups, with both features increasing with the severity of myopia (Table 1). There was no difference between groups in Humphrey visual field (HVF) mean deviation scores, average retinal nerve fiber layer (RNFL) thickness and cataract status (Table 1).

**Table 1:** Comparison of demographic and clinical characteristics between groups.

	Hyperopia	Emmetropia	Low Myopia	Moderate Myopia	*P* value	All Cases
Sample Size	54	31	30	24	—	139
Age (years)*	63.0 (8.0)^||^	59.0 (8.0)^||^	60.5 (12.0)	60.0 (10.3)	0.03^‡^	61.0 (9.5)
Gender (%males)	35.2	38.7	46.7	25	0.42^§^	36.7
Ethnicity (%Chinese)	79.6	90.3	90	95.8	0.19^§^	87.1
SE (D)	1.72 (1.0)	0.01 (0.3)	−1.66 (0.7)	−3.96 (0.8)	<0.001^†^	−0.37 (2.2)
AxL (mm)	23.23 (0.8)	23.78 (0.6)	24.32 (0.8)	25.34 (0.9)	<0.001	23.95 (1.1)
ACD (mm)	3.00 (0.4)^#^	3.03 (0.3)	3.21 (0.3)^#^	3.23 (0.3)^#^	0.004	3.09 (0.3)
VFMD (dB)*	−1.67 (2.6)	−1.14 (2.2)	−1.20 (1.7)	−0.94 (2.3)	0.18^‡^	−1.19 (2.4)
RNFL thickness (µm)	94.35 (8.8)	92.97 (9.6)	91.63 (8.1)	93.88 (9.6)	0.60	93.37 (9.0)
**Cataract status**					0.77^§^	
no cataract (%)	41.7	50.0	38.7	33.3		39.6
NS1 (%)	50.0	46.7	58.1	59.2		54.7
NS2 (%)	8.3	3.3	3.2	7.4		5.8

Abbreviations: ACD = anterior chamber depth; AxL = axial length; dB = decibels; SE = spherical equivalent; RNFL = retinal nerve fiber layer; VFMD = visual field mean deviation.

Data are represented as average (SD) when data were normally distributed or median (inter-quartile range) when data were not normally distributed^*^. Data were compared using a One-way analysis of variance (ANOVA) when data were normally distributed and showed homogenous variance or Welch ANOVA when data were normally distributed and showed heterogeneous variance^†^ or one-way ANOVA on ranks when data were not normally distributed^‡^. ^§^Statistics done using a χ^2^ test. When post hoc significance is not represented this implies that all groups were different pairwise. ^||^In the post hoc analysis, participants with hyperopia were significantly older than emmetropic participants (*P* < 0.05). ^#^In the post hoc analysis, the ACD of participants with low or moderate myopia was significantly increased compared to participants with hyperopia.

### Baseline pupil diameter increased with the severity of myopia

**Figure 1:**
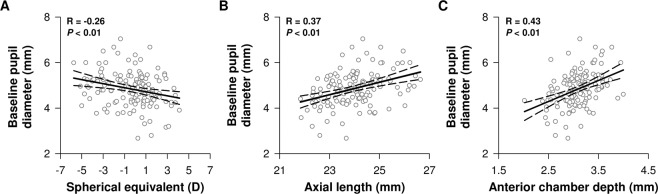
Correlations between baseline pupil diameter and clinical features of refractive error. Baseline pupil size assessed in dark conditions decreased as a function of spherical equivalent score (**A**) and was positively correlated with axial length (**B**) and anterior chamber depth (**C**).

Baseline pupil diameter, assessed in darkness prior to exposure to blue light, correlated inversely with spherical equivalent (*R* = −0.26, *P* < 0.01) (Fig. 1A), and positively with AxL and ACD (*R* = 0.37, *P* < 0.01 and *R* = 0.43, *P* < 0.01 respectively) (Fig. 1B,C). Corroborating the correlation analysis described above, baseline pupil diameter was significantly different between the four refractive error groups (F_3,135_ = 3.86, *P* = 0.01, Table 2). In post hoc analysis, only eyes with moderate myopia displayed a larger baseline pupil size in darkness compared to eyes with hyperopia (*P* = 0.02) (Table 2). Similar to blue light, baseline pupil diameter prior to red light exposure was also different between groups with eyes with moderate myopia displaying larger pupil size in darkness compared to eyes with hyperopia (*P* = 0.02) (Table 2). The amplitude of pupillary constriction in response to different irradiances of blue and red lights did not correlate with clinical features of refractive error (*i.e*., spherical equivalent, AxL, ACD) (Supplementary Fig. 1).

**Table 2:** Comparison of pupillometric outcome measures between groups.

	Hyperopia	Emmetropia	Low Myopia	Moderate Myopia	*P* value	All Cases
**Blue light**						
Baseline pupil diameter (mm)	4.60 (0.7)^‡^	4.84 (0.8)	5.00 (0.8)	5.16 (0.6)^‡^	0.01	4.84 (0.8)
Threshold irradiance (Log photons/cm²/s)*	11.34 (1.5)	11.41 (1.4)	11.78 (1.4)	11.55 (1.2)	0.30^†^	11.48 (1.4)
PIPR (%)^*^	20.32 (11.3)	20.37 (9.7)	21.44 (8.9)	23.58 (4.9)	0.49^†^	21.84 (9.4)
**Red light**						
Baseline pupil diameter (mm)*	4.65 (0.7)^‡^	4.85 (0.8)	5.05 (0.9)	5.21 (0.7)^‡^	0.01^†^	4.88 (0.8)
Threshold irradiance (Log photons/cm²/s)*	11.28 (1.5)	11.24 (1.4)	11.66 (0.9)	11.43 (1.1)	0.48^†^	11.41 (1.4)
PIPR (%)*	25.28 (8.8)	20.31(6.6)	23.58 (9.5)	26.97 (11.8)	0.05^†^	23.49 (9.6)
**Blue - Red**						
Difference in baseline pupil diameter prior to blue and red light exposure (mm)*	−0.09 (0.1)	−0.03 (0.1)	−0.08 (0.2)	−0.09 (0.2)	0.69^†^	−0.07 (0.2)

Abbreviations: PIPR = post-illumination pupillary response.

Data are represented as average (SD) when data were normally distributed or median (inter-quartile range) when data were not normally distributed*. Data were compared using a One-way analysis of variance (ANOVA) when data were normally distributed and showed homogenous variance or one-way ANOVA on ranks when data were not normally distributed^†^. ^‡^In the post hoc analysis, baseline pupil diameter prior to blue and red lights was significantly larger in eyes with moderate myopia compared to eyes with hyperopia (*P* < 0.05). There was no significant difference between other groups.

### Pupillary responses to blue light are not affected by low and moderate refractive error

**Figure 2:**
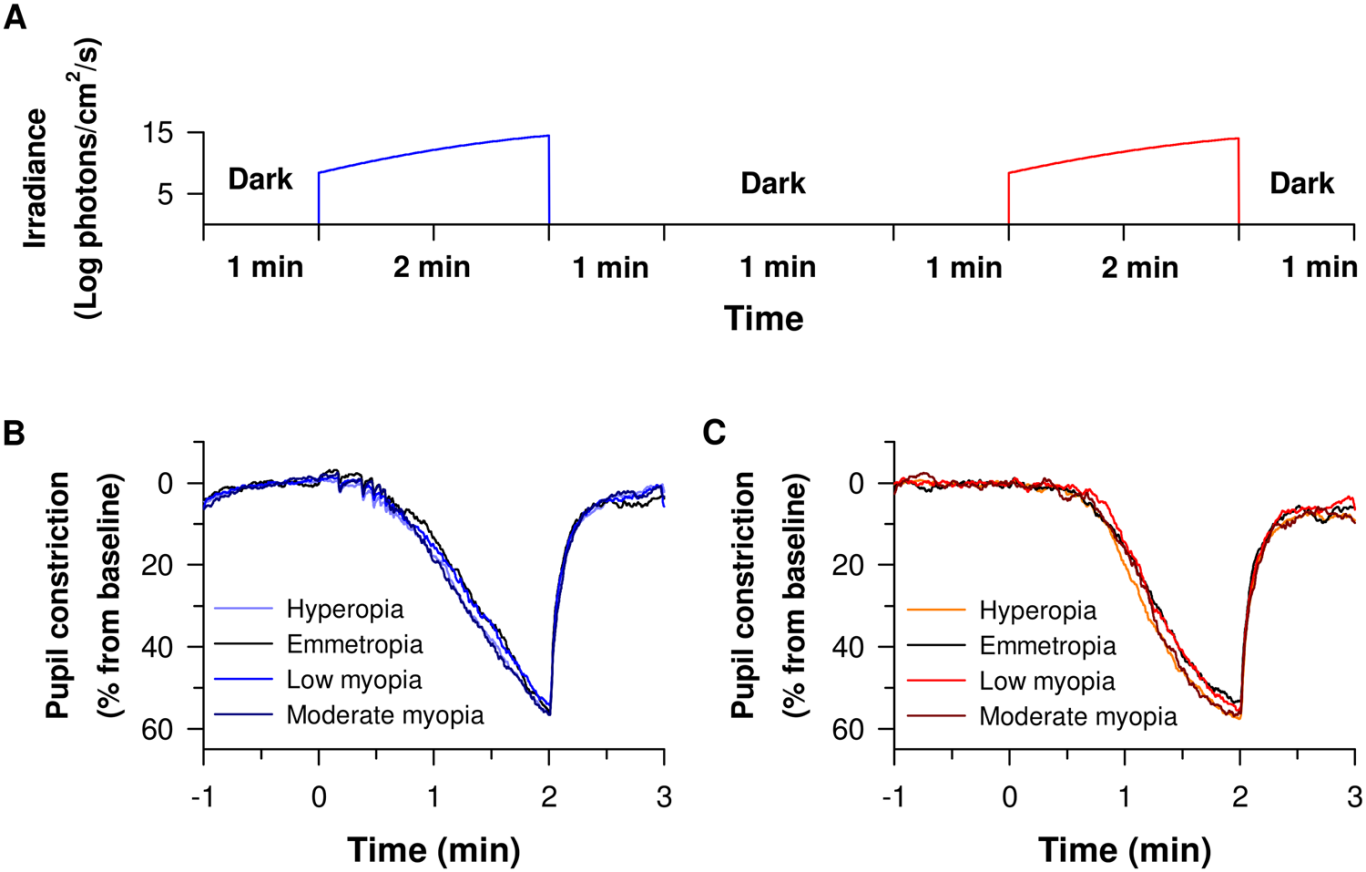
Experimental protocol and average pupillary constriction in response to blue and red lights in different study groups. (**A**) Each participant was exposed to logarithmically ramping-up (blue (462 nm, 8.5 to 14.5 Log photons/cm^2^/s) and red (638 nm, 8.5 to 14.0 Log photons/cm^2^/s) light stimuli. One minute of darkness preceded and followed each light exposure. One minute of darkness separated the blue and red light exposure protocols. Baseline-adjusted pupillary constriction responses of all study groups in response to ramping-up blue **(B)** and red light **(C)** exposures.

**Figure 3:**
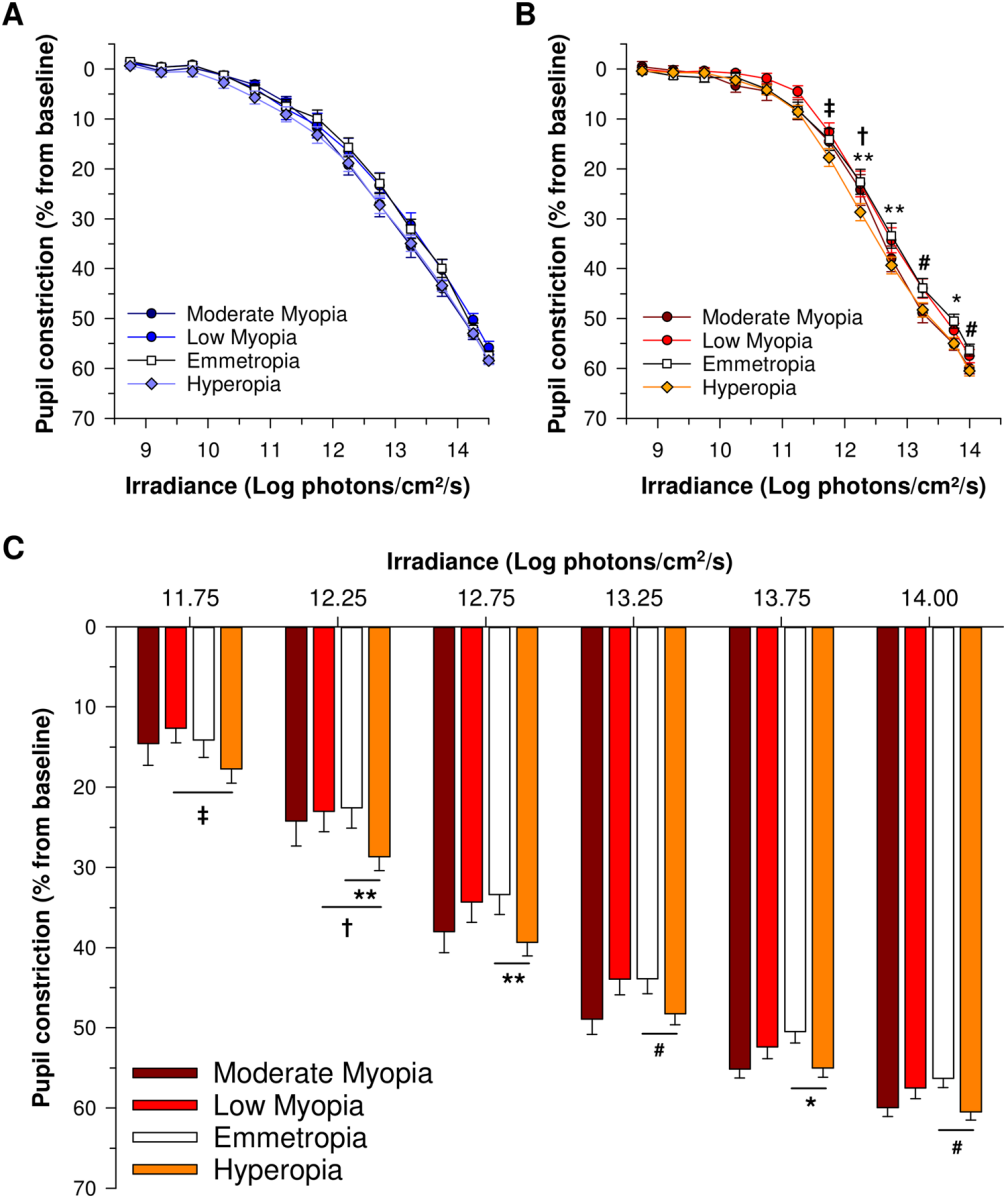
Irradiance-response curves to blue and red lights in the different study groups. Pupillary constriction amplitudes to ramping-up blue light did not differ between refractive error groups compared to emmetropes (**A**). Pupillary constriction amplitudes in response to red light was not different between myopia groups (low and moderate myopia) and emmetropes but was increased in hyperopes at moderate to high irradiances (≥12.25 Log photons/cm^2^/s) compared to emmetropes, and at moderate irradiances (11.75 to 12.25 Log photons/cm²/s) in hyperopes compared to low myopes (**B**,**C**). Panels A and B depict the irradiance response curves to blue and red lights in all study groups. Panel C depicts the average constriction responses of each group presented as bar plots between 11.75 and 14.0 Log photons/cm^2^/s for red light. Data are represented as average ± SE. For post hoc pairwise comparison between hyperopia and emmetropia groups *P < 0.05, **P < 0.01, ^#^P < 0.1. For post hoc pairwise comparison between hyperopia and low myopia groups ^†^P < 0.05, ^‡^P < 0.1.

**Figure 4:**
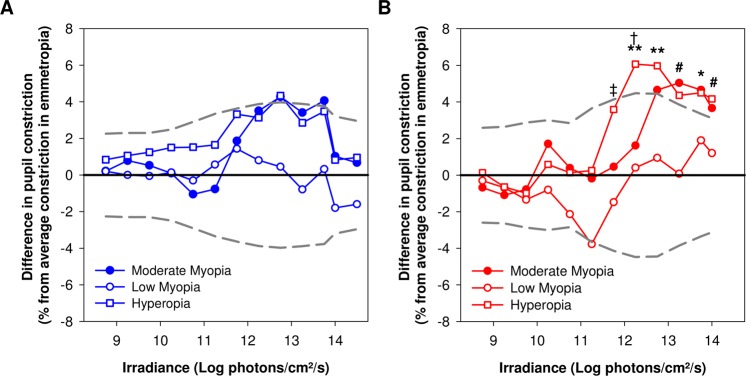
Difference in pupil constriction at different irradiances between refractive error groups and controls (emmetropia). (**A**) Pupillary constriction to a ramping-up blue light stimulus was not different from emmetropic controls in the 3 groups with refractive error. (**B**) Participants with hyperopia displayed an increase in constriction to moderate and high light intensities (≥12.25 Log photons/cm²/s) compared to emmetropes. The amplitudes of pupillary constriction were not significantly different between low and moderate myopes compared to emmetropes. Data are presented as average irradiance response curves of pupillary constriction of each group normalized to the emmetropic group by means of subtraction. The average pupillary constriction amplitude in emmetropes is shown here as a black full line. The 95% confidence interval of pupillary constriction amplitude in the emmetrope group is shown as grey dashed lines. Pupillary constriction amplitudes at different irradiances are either increased (+) or decreased (−) compared to emmetropes at different irradiances. Statistical comparisons reported in this figure are based on the LMM and post-hoc pairwise comparisons performed on baseline-adjusted irradiance response curves. For post hoc pairwise comparison between hyperopia and emmetropia groups *P < 0.05, **P < 0.01, ^#^*P* < 0.1. For post hoc pairwise comparison between hyperopia and low myopia groups ^†^*P* < 0.05, ^‡^*P* < 0.1.

**Figure 5:**
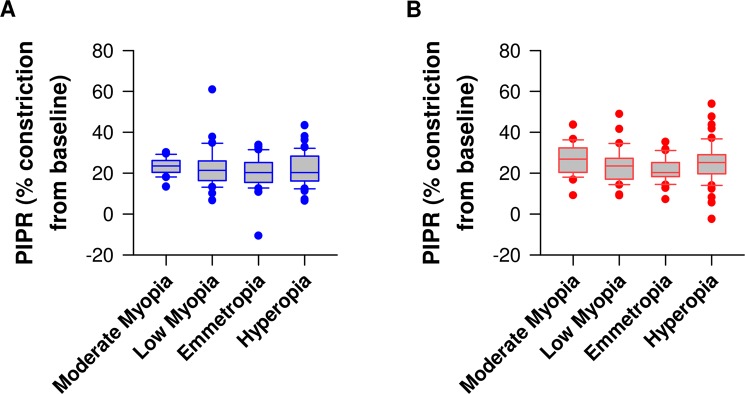
Post-illumination pupillary response (PIPR) to blue and red lights in different study groups. PIPR was not different between groups in response to blue light (**A**) and red light (**B**).

While the logarithmic increase in irradiance (Fig. 2A) led to a gradually increasing pupillary response to blue light (F_12,1446.9_ = 2018.4, *P* < 0.001) in all groups (Figs 2B, 3A), the amplitude of baseline-adjusted pupillary constriction was not different between refractive error groups (F_3,126.8_ = 0.89, *P* = 0.45) (Figs 3A, 4A, Supplementary Fig. 2) nor was it dependent upon the age of participants (*P* = 0.08). The threshold irradiances of constriction and PIPR in response to the blue light stimulus were not different between groups (Fig. 5A, Table 2).

### Pupillary constriction amplitudes to moderate and high levels of red light are increased in participants with hyperopia

The logarithmic increase in irradiance led to gradual pupillary constriction to red light (F_11,1263.5_ = 1559.6, *P* < 0.001) in all groups (Figs 2C and 3B). Age was a significant covariate (*P* = 0.001) and the amplitude of pupillary light constriction was not different between groups in general (F_3,140.1_ = 2.1, *P* = 0.11). However, the effect of irradiance on baseline-adjusted pupillary responses varied by group (irradiance × group interaction; F_33,1263.5_ = 1.5, *P* = 0.04) with patients with hyperopia displaying an increased pupil constriction amplitude to moderate and high light intensities (12.25 to 14.0 Log photons/cm²/s) compared to emmetropes (Figs 3B,C, 4B, Supplementary Fig. 2), and moderate light intensities compared to low myopes (11.75 to 12.25 Log photons/cm²/s) (Figs 3B,C, 4B, Supplementary Fig. 2). The amplitudes of pupillary constriction were not significantly different between low or moderate myopes compared to emmetropes (Figs 3C, 4B, Supplementary Fig. 2). The threshold irradiances of constriction and the PIPR in response to the red light stimulus were not different between groups (Fig. 5B, Table 2).

## Discussion

In this study, we found that ocular refractive status in healthy subjects affected baseline pupil diameter in darkness, with hyperopes having the smallest pupils and myopes the largest. Baseline-adjusted pupillary constriction amplitudes to ramping-up blue and red lights were not altered in eyes with low and moderate myopia compared with emmetropic eyes. However, pupillary constriction was greater in eyes with hyperopia compared to emmetropic eyes at moderate to high intensities of red light. Other pupillometric indices such as PIPR and threshold irradiance of constriction were not affected by ocular refractive status.

The increased baseline pupil size in darkness, observed with increasing severity of myopia in general, and between moderate myopic eyes and hyperopic eyes in particular, is consistent with previous studies performed in darkness and low light conditions^28,40^, but in disagreement with others^37,39^. There are several possible explanations for the larger pupil in myopic eyes. It is conceivable that due to the synkinesis between accommodation and pupil constriction, emmetropes and uncorrected hyperopes may accommodate more at a near visible target, than uncorrected myopes^41^. However, in our study, the increased baseline pupil size in myopes cannot be explained by sheer accommodation, as baseline pupil size was assessed in darkness without any visible near target that may trigger an accommodation reflex in participants. Another possible explanation for the larger baseline pupil diameter in moderate myopes may have been related to the global morphometric features of myopic eyes, compared to emmetropes. Myopic eyes have a longer axial length^42^, which may impact, as a consequence, the size of their pupil. Indeed, in our study increased axial length and larger anterior chamber associated with increasing degree of myopia and larger baseline pupil size.

While the dark-adapted pupil diameter is governed by a closed loop of autonomic control, the PLR also relies on the integrity of retinal photoreception^43^. Previous investigations of retinal function using multifocal electroretinogram (mfERG) have reported reduced amplitudes and delayed responses in myopic eyes (excluding high myopia) as compared to emmetropic eyes^44,45^. These findings were postulated to be secondary to cone dysfunction, damage in the inner plexiform layer or a delay in synaptic transfer from photoreceptors to bipolar cells. While the PLR induced by blue light at moderate to bright light intensities originates predominantly from the intrinsic response of melanopsin expressing retinal ganglion cells and thus bypasses any underlying outer-retinal or synaptic defects in myopic patients, such defects would have prompted an abnormal response to red light in patients with low and moderate myopia, as reported in patients with outer retinal diseases^19,46^. We observed normal pupillary responses to ramping-up red light in myopic patients, which does not support outer retinal or synaptic dysfunction in low or moderate myopia. These findings are in agreement with recent findings by Adhikari and colleagues^24^ in a small subset of hyperopic and myopic participants using a different light paradigm. Electrophysiological alterations in myopic eyes may essentially be due to anatomical changes (*e.g*., increased axial length) affecting electrical signal strength recorded at the corneal level. It is also plausible that chromatic pupillometry is not sensitive enough to detect mild sub-clinical retinal dysfunction occurring in low-to-moderate myopia.

In this study, we also report an increased amplitude of pupillary constriction to moderate to bright intensities of red but not blue light in hyperopic eyes. Hyperopic eyes exert greater levels of accommodation than emmetropic and myopic eyes, when no refractive error correction is worn^38^. Even though accommodation is not a major contributor to pupil diameter under white light^38^, a potential explanation to the wavelength-dependent increase in constriction observed in our study is the need for an increased accommodative reflex in hyperopes especially under red light focused behind the retina by virtue of longitudinal chromatic aberration (LCA)^47,48^. Conversely, blue light focused in the anterior part of the retina would require less accommodation in hyperopic eyes and thus less pupillary constriction. This is plausible in our study because the fixation target, appearing with the increasing light stimulation, was not presented at infinity and may have induced an accommodative reflex after light onset. Even though the influence of accommodation on pupil size in older individuals is expected to be small as compared to the effect of light intensity^49^, correcting for LCA using Atchison and Smith’s template for chromatic difference in refraction in this study^50^, eliminates differences in pupillary constriction responses to red light between hyperopes and emmetropes (results not detailed in this manuscript). Additional studies using pre- and post- refractive correction by contact lenses, are required to confirm the effect of hyperopia and elucidate the potential confounding effect of chromatic aberration and accommodation in chromatic pupillometry.

Recent studies have supported chromatic pupillometry as a potential non-invasive and objective clinical tool for assessment of retinal and optic nerve pathologies. Abnormal pupillometric parameters have been reported in optic neuropathies^14–16^, retinal dystrophies^17,18^, macular degeneration^20^, and diabetic retinopathy^21,51^. Using a ramping-up lighting paradigm, similar to that used in this study, our team has recently demonstrated that early-stage glaucoma is associated with reduced pupillary responses to both blue and red lights^11^. A potential future application of chromatic pupillometry is to aid in the diagnosis of glaucoma in myopic patients when other modalities such as the HVF or OCT are inconclusive^52,53^. This is especially important as the prevalence of glaucoma is higher in myopic patients^54,55^. By demonstrating that low and moderate refractive errors do not affect the pupillary responses to gradually increasing blue light, we suggest that chromatic pupillometry remains a potential screening and diagnostic tool for inner retina and optic nerve diseases in myopic patients and in populations with a high prevalence of myopia. When outer-retinal diseases are evaluated using ramping-up red-light stimuli, the refractive error of the patient’s eyes should be considered.

Our study has a few limitations. First, we recruited middle-aged to older participants who might have different ocular media transmittance as compared to younger participants^22,23^, therefore, our results may not be generalizable to all age-groups. However, ocular diseases, potentially detectable using chromatic pupillometry, are more prevalent in older age-groups and previous work from our group shows that mild to moderate cataracts does not affect the PLR using the ramping-up light protocol^26^. Second, we did not include patients with high myopia (spherical equivalent <−6D), since high myopia is associated with higher prevalence of confounding pathological complications like posterior staphyloma and chorioretinal atrophy^56^. Further study is warranted to investigate the exact impact of high myopia in the absence of pathology on chromatic pupillometry indices. Third, most of our participants were of Asian descent and therefore additional research is required before our findings can be generalized to other ethnicities. Finally, patient comfort may have been compromised by the relatively long duration (2 minutes) of the ramp-up stimuli used in this study and photoreceptor contribution to the PLR may have been blunted by the short duration of dark adaptation used in this study. While the aim of this study was to evaluate the effect of refractive errors on pupillary metrics over a wide range of light intensities using a full-field ramping-up light protocol, we have previously shown that using this light paradigm allows for 1) the construction of dose response curves over a large range of light intensities for both blue and red lights in the course of a single 2-minute exposure^8^; 2) the detection of pupillometric alterations in patients with early-stage glaucoma compared to controls^11^. Such alterations are not detected using a single full-field 1 s light exposure^12^ but can also be detected when intricate short-duration quadrant stimulations are used^13^.

In this study, we evaluated the effect of mild to moderate refractive errors on the direct pupillary responses to full-field ramping-up lighting protocols, in a large sample of older Asian participants. In conclusion, while myopia is associated with larger baseline pupil size in darkness, the pupillary response to a ramping-up blue light stimulus is not different between hyperopes, emmetropes and low to moderate myopes. More precaution might be needed in interpreting pupillary responses to red-light stimuli in hyperopic patients, as well as in those with higher degrees of refractive error.

## Methods

### Participants

One hundred and forty-eight participants aged 50 years or older were included in a cross-sectional study over a 15-month period (July 2015 to September 2016). The study took place at the Research Clinics of the Singapore Eye Research Institute (SERI). Participants were recruited from the general population through local advertisement and word-of-mouth referrals, had no previous or existing ophthalmic or general health conditions, nor were they on medications known to affect pupil size or pupillary responses to light.

All participants underwent a standardized ophthalmic evaluation which comprised slit lamp, fundus, and gonioscopic examination, best corrected visual acuity (BCVA) (LogMAR, Lighthouse, Inc., NY, USA) and color vision testing (Ishihara plates, Kanehara & Co., Tokyo, Japan), as well as auto-refraction (non-contact Auto Kerato-Refracto-tonometer TRK-1P, Topcon, Tokyo, Japan). Participants with spherical equivalent refractive error greater than +6.0D or less than −6.0D, spherical refractive error greater than +6.0D or less than −6.0D, or cylindrical refractive error greater than 3.0D, were excluded from the study. Participants who had undergone prior ocular surgery including those with pseudophakia, were also excluded from the study. AxL and ACD were measured using noncontact partial coherence laser interferomety (Lenstar LS900, Haag-Sgtreit AG, Switzerland). Subjects also underwent standard automated perimetry using the 24–2 Swedish Interactive thresholding algorithm with stimulus size III (Humphrey visual field Analyzer II model 750; Carl Zeiss Meditec, Dublin, CA). High definition optical coherence tomography (HD-OCT) (Cirrus version 6.0, Carl Zeiss Meditec, Dublin, CA, USA) was used to quantify the RNFL thickness. OCT results were validated only if the recorded signal strength had a value of 6 or better. The study was approved by the SingHealth Centralized Institutional Review Board, and written informed consent was obtained from all participants. Research procedures adhered to ethical principles outlined in the Declaration of Helsinki.

### Chromatic Pupillometry

Chromatic pupillometry was performed in all subjects using a protocol previously described^11,57^. Briefly, the direct PLR was assessed in one eye with the fellow eye occluded to avoid consensual interference. Horizontal pupil diameter was recorded continuously at a sampling rate of 120 frames per second using an infrared pupilometer (ETL-100H Pupillometry Lab; ISCAN Inc, Woburn, MA, USA). Participants were seated, without wearing any refractive correction, in complete darkness (<0.003 Lux), with their chin on a chin-rest, before being exposed to light *via* a modified Ganzfeld dome (Labsphere, Inc, North Sutton, NH, USA) equipped with narrow bandwidth light-emitting diodes (LED). The light exposure protocol consisted of 2 minutes of logarithmically increasing intensity of blue light (462 nm; 8.5 to 14.5 Log photons/cm^2^/s) followed by a similar exposure to red light (638 nm; 8.5 to 14.0 Log photons/cm^2^/s) measured at the cornea. (Fig. 2A). One minute of darkness preceded and followed each light exposure. One minute of darkness separated the blue and red light exposure protocols (Fig. 2A). During light exposure, participants were instructed to maintain a stable gaze and fixate a cross located at the center of the dome. Appropriate fixation was monitored in real-time by study personnel to avoid fixation losses. If fixation losses occurred frequently or the participant was unable to maintain fixation, the experiment was repeated. The Ganzfeld dome and chin-rest were surrounded by a dark curtain to ensure light isolation.

### Outcome measures from chromatic pupillometry

Horizontal pupil diameter measurements were processed for blink artefact removal and then expressed as a percentage change from baseline pupil size observed prior to each light exposure (*i.e*., blue or red) using the following equation:

$${\rm{Pupil}}\,\mathrm{constriction}\,( \% \,{\rm{from}}\,{\rm{baseline}})=100\times \frac{{\rm{Baseline}}\,{\rm{pupil}}\,{\rm{size}}-{\rm{Pupil}}\,{\rm{Size}}}{{\rm{Baseline}}\,{\rm{Pupil}}\,\mathrm{Size}\,}$$

Baseline pupil size was calculated as the median horizontal pupil diameter during the 30 seconds of darkness preceding each light exposure. Baseline-adjusted pupil constriction amplitudes were binned in 0.5 Log unit bins from 8.5 to 14.5 Log photons/cm^2^/s for blue light and 8.5 to 14 Log photons/cm^2^/s for red light. The median constriction response during each bin was determined and used to construct individual irradiance response curves. The maximum constriction response during each light exposure protocol was also determined and included in the irradiance response curves. Threshold of pupillary constriction was defined as the irradiance at which the pupil reached 10% of constriction from baseline. The post illumination pupil response (PIPR) was derived as the percent pupil constriction 6 seconds after blue or red light-offset, given that 6-seconds PIPR metrics yields lowest intra- and inter-individual variability^58^.

### Data analysis and statistics

The linear relationship between PLR features (*i.e*., baseline pupil diameter prior to blue-light exposure, threshold of constriction, PIPR, and baseline-adjusted constriction at different irradiances) and clinical features of refractive error (spherical equivalent, ACD, AxL) was assessed using Pearson’s correlation analysis. Welch analysis of variance (ANOVA) and Games-Howell Post-hoc tests were used to compare spherical equivalent scores between groups given the normal distribution and heterogeneous variance of the data. One-way ANOVA or ANOVA on ranks (for non-normally distributed variables) were used to compare baseline pupil size, threshold of constriction, PIPR and other clinical parameters between groups. Baseline-adjusted pupil constriction amplitudes were compared between groups and across light intensities using a linear mixed model analysis with irradiance and group as within- and between-subject factors respectively and age as co-variate. For those comparisons in which the omnibus test reached statistical significance, pairwise multiple comparison procedures were performed using the Holm-Sidak method or a Dunn’s test (for non-normally distributed variables). Normality of data distribution was determined using Shapiro-Wilk test. For all statistical tests other than correlation analyses, the threshold for significance was set at α = 0.05. A conservative threshold for significance of α = 0.01 was set to determine substantive evidence for correlation between features^59^. Data were analysed using MATLAB Release 2017, (The MathWorks, Inc., Natick, MA, USA), and SPSS Version 22.0 software (IBM Corp., Armonk, NY, USA). Figures were plotted using Sigmaplot 14.0 (Systat Software, Inc., San Jose, CA USA).

## Supplementary information

Supplementary information

## Data Availability

The datasets collected and analysed during the current study (eliminating identifying information) are available from the corresponding author on reasonable request.

## References

[1] Hattar S (2003). Melanopsin and rod-cone photoreceptive systems account for all major accessory visual functions in mice. Nature.

[2] Berson DM, Dunn FA, Takao M (2002). Phototransduction by retinal ganglion cells that set the circadian clock. Science.

[3] Guler AD (2008). Melanopsin cells are the principal conduits for rod-cone input to non-image-forming vision. Nature.

[4] Provencio I (2000). A novel human opsin in the inner retina. The Journal of neuroscience: the official journal of the Society for Neuroscience.

[5] Bailes HJ, Lucas RJ (2013). Human melanopsin forms a pigment maximally sensitive to blue light (lambdamax approximately 479 nm) supporting activation of G(q/11) and G(i/o) signalling cascades. Proc Biol Sci.

[6] Merbs SL, Nathans J (1992). Absorption spectra of human cone pigments. Nature.

[7] Kardon R (2009). Chromatic pupil responses: preferential activation of the melanopsin-mediated versus outer photoreceptor-mediated pupil light reflex. Ophthalmology.

[8] Rukmini AV (2015). Pupillary Responses to High-Irradiance Blue Light Correlate with Glaucoma Severity. Ophthalmology.

[9] Feigl B, Zele AJ (2014). Melanopsin-expressing intrinsically photosensitive retinal ganglion cells in retinal disease. Optometry and vision science: official publication of the American Academy of Optometry.

[10] La Morgia C (2011). Melanopsin-expressing retinal ganglion cells: implications for human diseases. Vision research.

[11] Najjar RP (2018). Pupillary Responses to Full-Field Chromatic Stimuli Are Reduced in Patients with Early-Stage Primary Open-Angle Glaucoma. Ophthalmology.

[12] Feigl B, Mattes D, Thomas R, Zele AJ (2011). Intrinsically photosensitive (melanopsin) retinal ganglion cell function in glaucoma. Investigative ophthalmology & visual science.

[13] Adhikari P, Zele AJ, Thomas R, Feigl B (2016). Quadrant Field Pupillometry Detects Melanopsin Dysfunction in Glaucoma Suspects and Early Glaucoma. Scientific reports.

[14] Moura AL (2013). The pupil light reflex in Leber’s hereditary optic neuropathy: evidence for preservation of melanopsin-expressing retinal ganglion cells. Investigative ophthalmology & visual science.

[15] Kawasaki, H & Sander, M Selective wavelength pupillometry in Leber hereditary optic neuropathy. *Clinical & experimental ophthalmology* (2010). 10.1111/j.1442-9071.2010.02212.x 20447133

[16] Tsika C, Crippa SV, Kawasaki A (2015). Differential monocular vs. binocular pupil responses from melanopsin-based photoreception in patients with anterior ischemic optic neuropathy. Scientific reports.

[17] Skaat A (2013). Pupillometer-based objective chromatic perimetry in normal eyes and patients with retinal photoreceptor dystrophies. Investigative ophthalmology & visual science.

[18] Chibel R (2016). Chromatic Multifocal Pupillometer for Objective Perimetry and Diagnosis of Patients with Retinitis Pigmentosa. Ophthalmology.

[19] Kardon R (2011). Chromatic pupillometry in patients with retinitis pigmentosa. Ophthalmology.

[20] Maynard ML, Zele AJ, Feigl B (2015). Melanopsin-Mediated Post-Illumination Pupil Response in Early Age-Related Macular Degeneration. Investigative ophthalmology & visual science.

[21] Park JC (2017). Pupillary responses in non-proliferative diabetic retinopathy. Scientific reports.

[22] Najjar RP (2016). Heterochromatic Flicker Photometry for Objective Lens Density Quantification. Investigative ophthalmology & visual science.

[23] Teikari P (2012). Refined flicker photometry technique to measure ocular lens density. J Opt Soc Am A Opt Image Sci Vis.

[24] Adhikari P, Pearson CA, Anderson AM, Zele AJ, Feigl B (2015). Effect of Age and Refractive Error on the Melanopsin Mediated Post-Illumination Pupil Response (PIPR). Scientific reports.

[25] Abbott KS, Queener HM, Ostrin LA (2018). The ipRGC-Driven Pupil Response with Light Exposure, Refractive Error, and Sleep. Optometry and vision science: official publication of the American Academy of Optometry.

[26] Rukmini AV, Milea D, Aung T, Gooley JJ (2017). Pupillary responses to short-wavelength light are preserved in aging. Scientific reports.

[27] Hirsch MJ, Weymouth FW (1949). Pupil size in ametropia. Journal of applied physiology.

[28] Guillon M (2016). The Effects of Age, Refractive Status, and Luminance on Pupil Size. Optometry and vision science: official publication of the American Academy of Optometry.

[29] Wang Q, Klein BE, Klein R, Moss SE (1994). Refractive status in the Beaver Dam Eye Study. Investigative ophthalmology & visual science.

[30] Katz J, Tielsch JM, Sommer A (1997). Prevalence and risk factors for refractive errors in an adult inner city population. Investigative ophthalmology & visual science.

[31] Lin LL (1999). Epidemiologic study of ocular refraction among schoolchildren in Taiwan in 1995. Optometry and vision science: official publication of the American Academy of Optometry.

[32] Wu HM (2001). Does education explain ethnic differences in myopia prevalence? A population-based study of young adult males in Singapore. Optometry and vision science: official publication of the American Academy of Optometry.

[33] Pan CW (2013). Prevalence of refractive errors in a multiethnic Asian population: the Singapore epidemiology of eye disease study. Investigative ophthalmology & visual science.

[34] Pan CW (2012). Variation in prevalence of myopia between generations of migrant indians living in Singapore. American journal of ophthalmology.

[35] Yoo YC, Kim JM, Park KH, Kim CY, Kim TW (2013). Refractive errors in a rural Korean adult population: the Namil Study. Eye (Lond).

[36] Porterfield, W. *A Treatise on the Eye, the Manner and Phaenomena of Vision: In Two Volumes*. (A. Miller at London and G. Hamilton and J. Balfour at Edinburgh, 1759).

[37] Winn B, Whitaker D, Elliott DB, Phillips NJ (1994). Factors affecting light-adapted pupil size in normal human subjects. Investigative ophthalmology & visual science.

[38] Orr JB, Seidel D, Day M, Gray LS (2015). Is Pupil Diameter Influenced by Refractive Error?. Optometry and vision science: official publication of the American Academy of Optometry.

[39] Truong JQ, Joshi NR, Ciuffreda KJ (2018). Influence of refractive error on pupillary dynamics in the normal and mild traumatic brain injury (mTBI) populations. J Optom.

[40] Cakmak HB, Cagil N, Simavli H, Duzen B, Simsek S (2010). Refractive error may influence mesopic pupil size. Curr Eye Res.

[41] Jones R (1990). Do women and myopes have larger pupils?. Investigative ophthalmology & visual science.

[42] Yin G (2012). Ocular axial length and its associations in Chinese: the Beijing Eye Study. PloS one.

[43] Park JC (2011). Toward a clinical protocol for assessing rod, cone, and melanopsin contributions to the human pupil response. Investigative ophthalmology & visual science.

[44] Kawabata H, Adachi-Usami E (1997). Multifocal electroretinogram in myopia. Investigative ophthalmology & visual science.

[45] Chen JC, Brown B, Schmid KL (2006). Delayed mfERG responses in myopia. Vision research.

[46] Gooley JJ (2012). Melanopsin and rod-cone photoreceptors play different roles in mediating pupillary light responses during exposure to continuous light in humans. The Journal of neuroscience: the official journal of the Society for Neuroscience.

[47] Fincham EF (1951). The accommodation reflex and its stimulus. The British journal of ophthalmology.

[48] Seidemann A, Schaeffel F (2002). Effects of longitudinal chromatic aberration on accommodation and emmetropization. Vision research.

[49] Mathur A, Gehrmann J, Atchison DA (2014). Influences of luminance and accommodation stimuli on pupil size and pupil center location. Investigative ophthalmology & visual science.

[50] Atchison DA, Smith G (2005). Chromatic dispersions of the ocular media of human eyes. J Opt Soc Am A Opt Image Sci Vis.

[51] Ortube MC (2013). Comparative regional pupillography as a noninvasive biosensor screening method for diabetic retinopathy. Investigative ophthalmology & visual science.

[52] Ding X (2016). Visual field defect classification in the Zhongshan Ophthalmic Center-Brien Holden Vision Institute High Myopia Registry Study. The British journal of ophthalmology.

[53] Leung CK (2012). Retinal nerve fiber layer imaging with spectral-domain optical coherence tomography: interpreting the RNFL maps in healthy myopic eyes. Investigative ophthalmology & visual science.

[54] Mitchell P, Hourihan F, Sandbach J, Wang JJ (1999). The relationship between glaucoma and myopia: the Blue Mountains Eye Study. Ophthalmology.

[55] Xu L, Wang Y, Wang S, Wang Y, Jonas JB (2007). High myopia and glaucoma susceptibility the Beijing Eye Study. Ophthalmology.

[56] Curtin BJ, Karlin DB (1970). Axial length measurements and fundus changes of the myopic eye. I. The posterior fundus. Transactions of the American Ophthalmological Society.

[57] Rukmini AV (2017). Pupillary responses to light are not affected by narrow irido-corneal angles. Scientific reports.

[58] Adhikari P, Zele AJ, Feigl B (2015). The Post-Illumination Pupil Response (PIPR). Investigative ophthalmology & visual science.

[59] Sanfilippo PG, Casson RJ, Yazar S, Mackey DA, Hewitt AW (2016). Review of null hypothesis significance testing in the ophthalmic literature: are most ‘significant’ P values false positives?. Clin Exp Ophthalmol.

