# Supplementary material for: Effects of low and moderate refractive errors on chromatic pupillometry
Source: Sci Rep. 2019 Mar 20;9:4945. doi: 10.1038/s41598-019-41296-w (PMC6426861; doi:10.1038/s41598-019-41296-w)
Supplement: Supplementary file 1 — Supplementary information [file 41598_2019_41296_MOESM1_ESM.docx]

**Effects of low and moderate refractive errors on chromatic pupillometry**

A. V. Rukmini^╪^, Milton C. Chew^╪^, Maxwell Finkelstein, Eray Atalay, Mani Baskaran, Monisha E. Nongpiur, Joshua J. Gooley, Tin Aung, Dan Milea^*^, Raymond P. Najjar^*^

^╪^ Equal contribution

* Corresponding authors

**Supplementary material**

**Supplementary Figure 1**


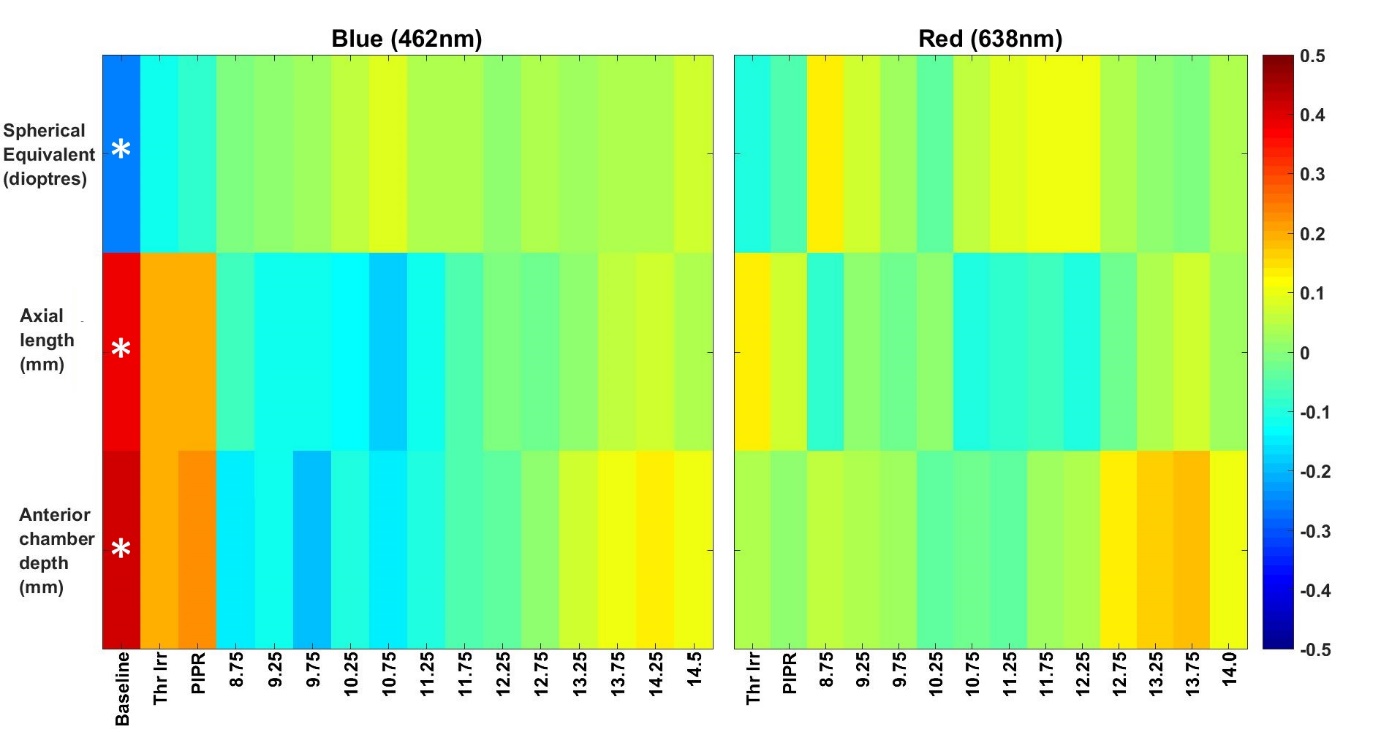


**Supplementary Figure 1**. **Correlations between pupillometric outcomes and ophthalmic features of refractive error.** The heat map shows Pearson’s correlation coefficient values for spherical equivalent, axial length and anterior chamber depth (y-axis) against baseline pupil size (Baseline), threshold irradiance for constriction (Thr Irr), post illumination pupillary response at 6sec (PIPR) and constriction amplitude at different irradiances of blue light (462nm) and red light (638nm). Dark blue indicates significant negative correlation and dark red indicates significant positive correlation. Only baseline pupil size correlates with the clinical measures, as seen in Figure 1. * : *P* < 0.05.

**Supplementary Figure 2**

**Supplementary Figure 2. Irradiance-response curves to blue and red lights in the different study groups.** Responses to blue light did not differ between groups with refractive errors compared to emmetropes (A, B). Pupillary constriction in response to red light was not different between myopia groups and emmetropes (C) but was increased in hyperopes at moderate to high irradiances (≥12.25 Log photons/cm^2^/s) compared to emmetropes (D), and at moderate irradiances (11.75 to 12.25 Log photons/cm²/s) in hyperopes compared to low myopes (not shown here). Data are represented as average ± SE. * : *P* < 0.05; ** : *P* < 0.01, # : *P* < 0.1.
